# Preparation and identification of a single domain antibody specific for adenovirus vectors and its application to the immunoaffinity purification of adenoviruses

**DOI:** 10.1186/s13568-022-01422-w

**Published:** 2022-06-20

**Authors:** Yi Cheng, Yanxia Hao, Fuxiang Bao, Huimin Zhang, Yanlong Liu, Kexin Ao, Shan Fu, Qiyao Wu, Zhi Wang

**Affiliations:** 1 College of Veterinary Medicine, Inner Mongolia Agricultural University, No. 29, Erdos East Street, Saihan District, Huhhot, 010010 Inner Mongolia China; 2 Key Laboratory of Clinical Diagnosis and Treatment Techniques for Animal Disease, Ministry of Agriculture and Rural Affairs (LDTA), Huhhot, China

**Keywords:** Adenovirus, Single domain antibody, Phage display library, Immunofluorescence, Immunoaffinity purification

## Abstract

Adenovirus belongs to the family of Adenoviridae. As a vaccine carrier, it has high safety and stimulates the body to produce cellular immunity and humoral immunity. This study prepared an adenoviral vector-specific single-domain antibody for use in adenovirus identification and purification. We successfully constructed a single domain antibody phage display library with a capacity of 1.8 × 10^9^ by immunizing and cloning the VHH gene from Bactrian camel. After the second round of biopanning, clones specific for adenovirus were screened using phage ELISA. Twenty-two positive clones were obtained, and two clones with the highest binding affinity from ELISA were selected and named sdAb 5 and sdAb 31 for further application. The recombinant single-domain antibody was solublely expressed in *E. coli* and specifically bound to adenoviruses rAd26, ChAd63 and HAd5 in ELISA and live cell immunofluorescence assays. We established an effective method for immunoaffinity purification of adenovirus by immobilizing the single domain antibody to Sepharose beads, and it may be used to selectively capture adenoviruses from cell culture medium. The preparation of the adenovirus-specific single-domain antibody lays a foundation for the one-step immunoaffinity purification and identification of adenoviruses.

## Introduction

Adenovirus (Ad) is a promising vector platform that may be used for vaccine development due to its high safety and stimulation of robust cellular response and humoral immunity in multiple species (Antrobus et al., 2014; Barnes et al., 2012; Coughlan et al., 2018). Adenovirus belongs to the family of Adenoviridae, and it is primarily isolated from five major vertebrates, including birds, mammals, reptiles, amphibians and fish. Approximately 200 non-human Ad serotypes have been identified. The diameter of the adenovirus shell is 70–90 nm, and it is composed of three main structural proteins: fiber, the penton base and the hexon. The genome of adenovirus is linear double-stranded DNA without an envelope (Trentin et al., 1962). Adenovirus stimulates the body's natural immune response without requiring immune adjuvants (Molinier-Frenkel et al., 2002). It has a broad spectrum of host cell tropism and infects host cells regardless of cell division (Chang, 2021). Adenoviruses exhibit some desirable characteristics. For example, their genomes are stable and easy to manipulate, which makes them particularly suitable for the development of preventive vaccines. These viruses may be amplified to produce high titers using a variety of complementary cell lines that comply with Good Clinical Practice (GCP) (Kovesdi and Hedley, 2010). They are now used as safe immunogenic vaccine vectors in clinical trials and exhibit outstanding performance (Gurwith et al., 2013; Liebowitz et al., 2020; Sebastian and Lambe, 2018).

Researchers modified the adenovirus genomes for safe and effective use in animals and humans. Deletion of the *E1/E3* region to obtain the first-generation adenovirus vector allows the insertion of a transgene with replication-defective properties and improved immunogenicity characteristics (Bett et al., 1994). However, the first-generation adenovirus vector genome is homologous to the *E1* region inserted into the packaging cell line HEK293, which may result in replicating-competent adenovirus (RCA). Researchers solved this problem using minimal homology cell lines (Kovesdi and Hedley, 2010). Second-generation adenoviral vector research found that additional deletion of the *E2/E4* site can increased transgenic ability and decreased the possibility of RCA formation. However, the second-generation adenoviral vector had decreased replication ability in producer cell lines (Wang and Finer, 1996), and the overall production yield of adenovirus vectors was lower than the first-generation adenovirus vectors. Third-generation adenoviral vectors, known as helper-dependent gutless adenoviral vectors, were obtained by deleting most genomic sequences except the regions that were necessary for packaging (Kochanek et al., 1996; Parks et al., 1996). Therefore, multiple transgene expression cassettes may be inserted, but with increasing difficulty of manufacturing. The immunogenicity of these vectors is also reduced due to deletion of the sequence, and there is a possibility of helper virus contamination.

When adenovirus is used as a vaccine carrier, its prevalence in humans must be considered during the research process. Pre-existing immunity reduces the effectiveness of the vaccine. Studies found that the globally most prevalent adenovirus vectors, human adenovirus serotypes 2 (HAd2) and HAd5, demonstrated variable results in clinical studies, and approximately 80% of the population had neutralizing antibodies against HAd5 (Farina et al., 2001). Therefore, scientists have tried to prepare adenovirus vectors using human adenovirus serotypes with low prevalence in the population and searched for adenoviruses of other species, such as chimpanzees and rhesus monkeys.

The prevalence of these alternative Ads is very low compared to Ad2 and Ad5 vectors, like Group D adenoviruses (such as Ad26, Ad48, Ad24, and Ad49). Neutralizing antibodies against Ad26 only exist in individuals in South Africa and Southeast Asia, and Ad5-specific neutralizing antibodies were detected at very high levels in all regions (Shenk, 1996). Compared with rhesus monkeys vaccinated with Ad5, rhesus monkeys vaccinated with Ad26 induced higher levels of interferon-γ (IFN-γ), interleukin-1 receptor agonist (IL-1RA), IL-6 and inducible protein-10 (IP-10) cytokines. Therefore, rAd26 played a better role in the field of vaccine research and development.

Researchers also isolated adenovirus from non-human species to perform clinical applications (Lewis and Rowe, 1970). The main purpose of using adenoviruses from non-human species is to avoid existing immunity to human adenovirus serotypes. The diseases caused by non-human adenoviruses are species specific, so these viruses do not harm humans. Studies showed that the use of chimpanzee adenoviruses (ChAd3, ChAd6, ChAd7, ChAd63, and ChAd68) in clinical trials in mice, non-human primates and humans demonstrated that these adenovirus vectors evaded the specific immunity of human adenovirus serotypes (Wold and Toth, 2013). These results identified ChAd63 as a good candidate for gene transfer applications (Ison and Hayden, 2016).

Adenovirus purification is performed using cesium chloride (CsCl) density gradient centrifugation. A density gradient is created when CsCl is placed in a strong centrifugal field, and the virus in CsCl is centrifuged to equilibrium for isolation. Viruses are collected according to buoyant density (Burova and Ioffe, 2005). This method is relatively well established and widely used in the laboratory. However, it has certain limitations, such as requiring several ultra-centrifugations to obtain the pure product, and it is not suitable for mass production. The recovery rate is only 10%-30%, and the purified viral activity is not stable. This traditional method no longer meets the demands, and many new methods were developed in recent years.

Lee et al. (Lee et al., 2009) combined anion-exchange chromatography and immobilized metal affinity membrane chromatography for the purification of recombinant adenovirus. The adenovirus was purified from clarified infectious lysate via anion-exchange chromatography using Q Sepharose XL resin. The virus was further purified using a Sartobind IDA membrane unit charged with Zn2 + ions as affinity ligands, which increased the yield of adenovirus purification significantly. Peixoto et al. (Peixoto et al., 2006) reported a novel expanded bed chromatography method to purify adenovirus vectors. This technology is characterized by expanded beds concentrated by hollow fiber cartridges that harvest viral particles from cell extracts directly. For a minimal amount of sample handling, the purification is completed in less than one working day, and the overall process yield reaches 32%. However, this method remains difficult to scale up.

Single domain antibodies have only one heavy chain variable region domain called the variable domain of the heavy chain antibody (VHH), which are also referred to as nanobodies, and are extracted from Camelidae serum. These VHHs tightly bind to specific antigens. The relative molecular weight of nanobodies is only approximately 15 kDa, which allows them to have a strong tissue penetration ability (Hu et al., 2017). Nanobodies have good stability, may be stored for a long time at − 80 °C, and withstand high temperature, high pressure and extreme pH environments (Salvador et al., 2019). Therefore, nanobodies may be stockpiled as a therapeutic treatment option in the case of an epidemic. The simple molecular structure makes it easier to express in large quantities in yeast, *E. coli* and other microbial expression systems, which allows for industrialized large-scale production (Lafaye and Li, 2018). Compared to traditional antibodies, nanobodies do not have an Fc segment, and the sequence information encoding VHH is highly homologous to human VH families 3 (Ahmadvand et al., 2008), which exhibits very low immunogenicity in humans (Vincke et al., 2009). Part of the hydrophobic amino acids of nanobodies are replaced by hydrophilic amino acids, which improves solubility (Beghein and Gettemans, 2017). The CDR1 and CDR3 of nanobodies are longer than the conventional monoclonal antibody VH, which penetrates into the interior of the antigen and has better binding activity to the concave structure of the antigen (Padlan, 1996). Based on these excellent characteristics, nanobodies have been used as therapeutic and diagnostic agents in multiple research and clinical trials (Beghein and Gettemans, 2017).

The present study screened an antibody library for single domain antibodies that were specific for the adenovirus vector for coupling to the NHS-activated sepharose particles and use as an immunoaffinity purification ligand. This study provides a simple one-step adenovirus purification method from the cell culture medium that is relatively fast and efficient.

## Materials and methods

### Bactrian camel immunization

A healthy 3-year-old female Bactrian camel was selected for immunization with adenoviruses rAd26 and ChAd63 (gift of Professor Jinsheng He from Beijing Jiaotong University). The adenoviruses were diluted from the original concentration of 1 × 10^13^ vp/ml to 2 × 10^11^ vp/ml and 1 × 10^9^ vp/ml for immunization. Five milliliters of blood was collected from the jugular vein and coagulated naturally before immunization. The serum was collected and stored at − 20 °C. The camel was immunized four times, and the interval between immunizations was two weeks. One week after each immunization, blood was collected from the jugular vein and placed at 4 °C to separate the serum used for ELISA to detect the antibody titer. Two weeks after the fourth immunization, 50 ml of blood was collected from the jugular vein of the immunized camel for the separation of peripheral blood lymphocytes. The camel was farmed in a pasture located in the suburb of Hohhot city, Inner Mongolia and had free access to water and food. All experimental procedures were performed in accordance with the institutional and national guidelines and regulations and were approved by the Animal Care and Use Committee of Inner Mongolia Agriculture University.

### Construction and screening of Bactrian camel phage display antibody library

**Fig. 1:**
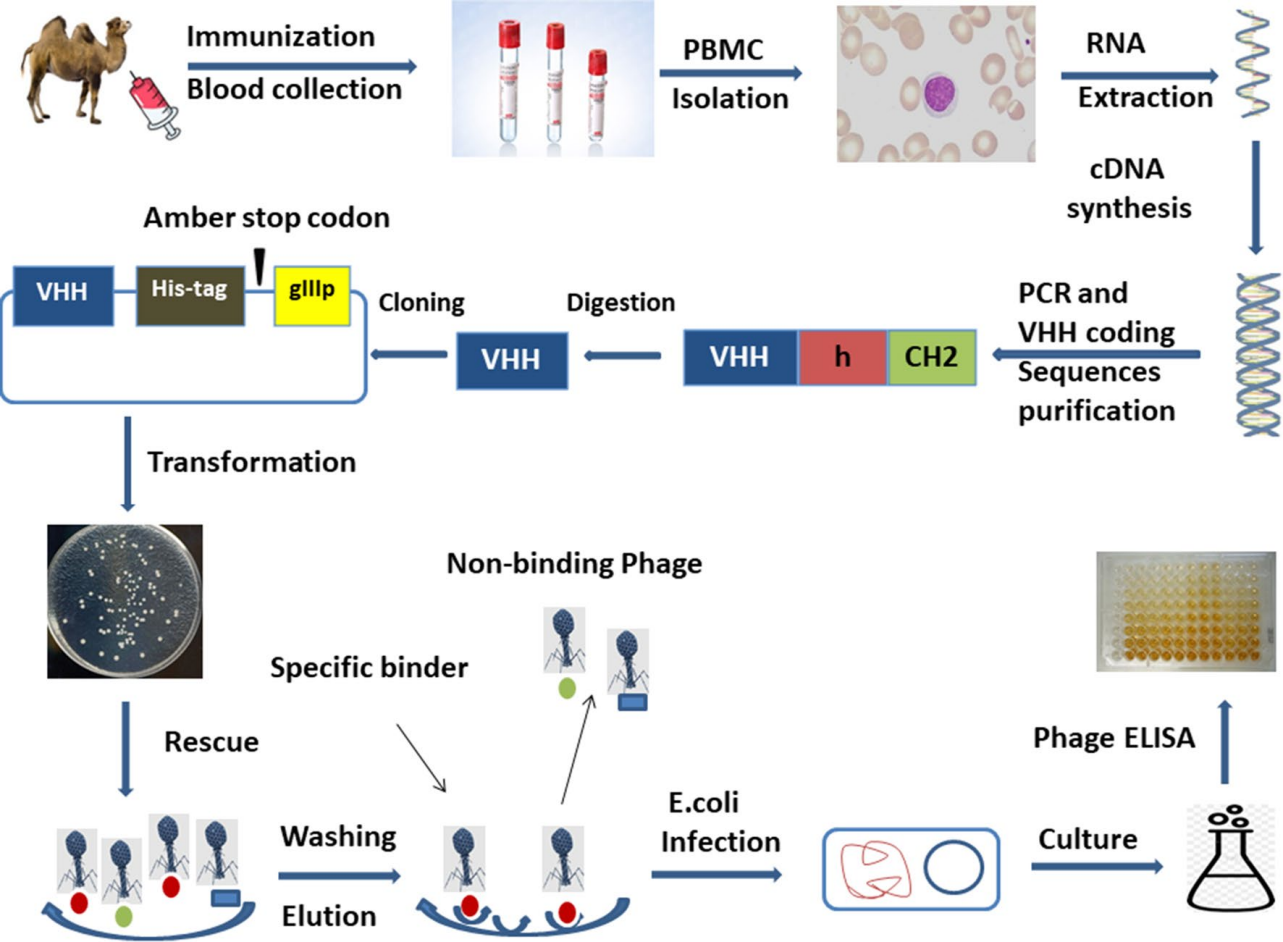
Schematic diagram of the construction and screening of a single-domain phage display library. The Bactrian camel was immunized with adenoviruses rAd26 and ChAd63, and the blood was collected to isolate peripheral blood mononuclear cells (PBMCs). Total RNA was extracted from PBMCs and reverse transcribed into cDNA. The VHH gene fragments were amplified using PCR. In the first round of PCR, a fragment of approximately 600 bp from the leader sequence to the CH2 domain was amplified from the cDNA. The full-length VHH gene from FR1 to FR4 was amplified using P_3_ and P_4_ primers in the second round of PCR. The PCR-amplified product was digested using the restriction enzymes *Nco*I and *Not*I*.* The VHH gene was ligated into the pMECS plasmid and transformed into *E. coli* TG1. After rescue by the M13k07 helper phage, the VHH gene was displayed on the phage surface, and the specific phages were enriched and selected using phage ELISA

The construction and screening of a single-domain phage display library are shown in Fig. 1. Peripheral blood monocytes (PBMCs) were prepared using an animal blood lymphocyte separation kit (TBD Science, Tianjin Haoyang Biological Products Technology Co. Ltd. China). TRIzol reagent (Ambion, USA) was used to extract total RNA from PBMCs, and RNA was reverse transcribed to cDNA using a reverse transcription kit (Promega, USA) and random hexamers as primers. The VHH fragment was amplified using the nested PCR method and the primers listed in Table 1. The first round of PCR used P_1_ and P_2_ as primers to amplify the antibody gene sequence from the leader peptide to the CH2 region from the cDNA. The PCR products were run on an agarose gel, and the VHH-containing band (600 bp) was recovered by cutting and extracting from the gel and used as the template for the second round of PCR. The full-length VHH gene from FR1 to FR4, with a fragment size of approximately 400 bp, was amplified from the second round of PCR using P_3_ and P_4_ as primers. The PCR-amplified product was digested using the restriction enzymes *Nco*I and *Not*I (New England Biolabs, UK), ligated into the pMECS plasmid vector (a gift from Professor Serge Muyldermans of the Vrije Universiteit Brussel, Belgium) with T4 ligase (New England Biolabs, UK), and transformed into *E. coli* TG1 competent cells (GE Healthcare, USA) to construct the antibody library. The capacity of the antibody library was estimated by calculating the colony forming units (CFU) of 10 series of multiple dilutions on a 2 × YT-AG plate. The transformation efficiency and the insertion of VHH were evaluated using PCR with the sequencing primers of the pMECS plasmid vectors MP57 and GIII, as listed in Table 1.

**Table 1:** The primers

Primers	Sequences	Products
P_1_	5'-GGTACGTGCTGTTGAACTGTTCC-3'	600 bp, 900 bp
P_2_	5'-GTCCTGGCTGCTCTTCTACAAGG-3'	
P_3_	5'-AGTTGTTCCTTCTATGCGGCCCAGCCGGCCATGGCTGAKGTBCA GCTGGTGGAGTCTGG-3'	
P_4_	5'-ATTGCGTCAGCTATTAGTGCGGCCGCTGAGGAGACRG TGACCWGGGTCC-3'	400 bp
MP57	5'-TTATGCTTCCGGCTCGTATG-3'	
GIII	5'-CCACAGACAGCCCTCATAG-3'	600 bp

The antibody library was added to 200 ml of 2 × YT medium, and the OD600 nm value of the bacterial solution did not exceed 0.3. The library was cultured in a shaker at 37 °C and 250 r/min for 2 h. The M13K07 helper phage (GE Healthcare, USA) was added and incubated at 37 °C for 1 h for infection. The cultured bacterial solution was centrifuged at 2000×*g* for 10 min at 4 °C. The supernatant was discarded, resuspended, and inoculated into 2 × YT-AK medium containing ampicillin and kanamycin at 37 °C and 220 r/min shaking overnight. The cultured medium was centrifuged at 4 °C and 7197×*g* for 15 min the next day, and the supernatant was added to 20% PEG8000 and centrifuged at 7197×*g* at 4 °C for 25 min to precipitate the phage. The supernatant was discarded, and the pellet was resuspended in PBS to obtain the recombinant phage library. The phage library was added to a 96-well microtiter plate coated with the adenovirus rAd26 at a concentration of 2 × 10^7^ vp/ml and bound at 37 °C for 1 h. The bound phage was eluted with 100 μl 100 mM triethylamine, sealed, incubated at room temperature for 10 min, and neutralized with 50 μl 1 M Tris–HCl. The eluted recombinant phage was used to infect *E. coli* TG1 (OD600 nm = 0.6), and M13K07 helper phage was added for rescue. The phages were harvested, purified and used for a new round of enrichment. The *E. coli* TG1 infected with the eluted phages from the final enrichment were grown on 2 × YT-AG plates. Ninety-two clones were randomly selected from the 2 × YT-AG plate, added to the 2 × YT-AG liquid medium, and incubated at 37 °C and 250 r/min overnight. The M13K07 helper phage was added and infected for 1 h. After centrifugation at 14,000 r/min for 5 min, the bacteria were resuspended in 2 × YT-AK medium and incubated at 37 °C and 250 r/min overnight. The medium was centrifuged at 14,000 r/min for 5 min, the supernatant was collected, 20% PEG8000 was added, and the mixture was centrifuged at 7197×*g* for 20 min. The recombinant phage was added to an ELISA plate precoated with 2 × 10^7^ vp/ml adenovirus rAd26, and the M13K07 helper phage was used as a negative control. PBS buffer was used as a blank control and bound for 2 h at room temperature. HRP/anti-M13 monoclonal conjugate (GE Healthcare, USA) diluted at 1:5000 was added to each well and bound for 1 h at room temperature. TMB solution was added for color development, and the OD450 nm value was determined. The absorbance of the experimental group/negative control ≥ 2.1 was regarded as positive.

### Expression and purification of sdAb

Plasmids of the positive clones were isolated from the phage-ELISA and double digested using the restriction enzymes *Nco*I and *Not*I. The digested VHH fragments were ligated into the pET-25b ( +) –SBP plasmid and digested using the same restriction endonuclease with T4 DNA ligase. The pET-25b ( +) vector carried a streptavidin-binding protein that was constructed and preserved by the Public Health Department of the School of Veterinary Medicine, Inner Mongolia Agricultural University. The products were transformed into *E. coli* BL21 (DE3) competent cells (TransGen Biotech, Beijing, China). The expression of recombinant sdAb was induced using isopropyl-β-d-thiogalactoside (IPTG) (Solarbio Life Sciences, Beijing, China) for 8–12 h. The cells were collected and sonicated. The precipitate and supernatant were collected and analyzed using SDS-PAGE. Ni–NTA Sefinose™ Resin (Sangon Biotech, Shanghai, China) was used to purify the expressed recombinant single domain antibodies, and the purified sdAbs were analyzed using SDS-PAGE.

### Binding activity and specificity of sdAb

To determine the binding activities of the purified recombinant sdAbs, the purified clone 5 and 31 sdAbs were diluted to a concentration of 5 μg/ml and added as a primary antibody to a 96-well plate coated with adenoviruses rAd26, ChAd63 and HAd5 at a concentration of 2 × 10^7^ vp/ml. Sonicated *E. coli* BL21 (DE3) transformed with empty pET-25b ( +) vector was used as a negative control, and PBS buffer was used as a blank control. Meanwhile, the purified clone 5 sdAbs were serial diluted from 30 μg/ml to 0.00152415 μg/ml to use as primary antibody and added to another 96-well plate coated with adenoviruses rAd26, ChAd63 and HAd5 at a concentration of 2 × 10^7^ vp/ml. HRP-conjugated 6*His-tag mouse monoclonal antibody (Proteintech Group, Wuhan, China) was used as the secondary antibody at a concentration of 1:10,000, and TMB solution was added for color development. The OD450 nm value was determined using a microplate reader.

### Binding affinity of recombinant sdAb

The affinity constants of purified clone 5 sdAbs to adenovirus rAd26 were determined by competitive ELISA. Adenovirus rAd26 at a titer of 2 × 10^7^ vp/ml was used to coat the 96-well plates overnight at 4 °C. The stock solution of adenovirus rAd26 was serial diluted from 2 × 10^9^ vp/ml to 2 × 10^2^ vp/ml, and mixed with 5 μg/ml of purified clone 5 sdAbs in a separate 96-well plates 1 h at 37 °C. The mixture was added to the antigen-coated wells and incubated for 2 h at 37 °C. After PBST washing, 100 μl of HRP-labeled anti-6*His-tag (1:10,000) was added to each well and incubated for 1 h at 37 °C. The wells were washed with PBST, and TMB solution was added for color development. The OD450 nm value was determined using a microplate reader. Affinity constants were expressed as the reciprocal of the adenovirus titer at 50% of the maximum OD450 nm value.

### Immunofluorescence assay

To improve cell adherence to the plate, the cell culture plate was treated with polylysine. One microliter per well of polylysine was added to a 24-well cell culture dish and incubated for 10 min. The liquid was discarded, and the cells were dried on a clean bench. HEK293A cells were incubated with 10^5^ cells/well in a polylysine-treated 24-well cell culture dish at 37 °C and 5% CO_2_ for approximately 24 h. The cell culture medium was discarded, and 1 ml of DMEM was added to each well. The adenovirus rAd26 was diluted to a concentration of 2 × 10^7^ vp/ml, 100 μl of virus diluent was added to each well (DMEM was added to the control group), and the cells were cultured in an incubator for 24 h. The medium was discarded, and 1 ml of paraformaldehyde was added to each well to fix the cells for 20 min at room temperature. A volume of 100 μl 0.3% Triton X-100 was added dropwise to each well, incubated at room temperature for 20 min, and rinsed with PBS 3 times for 5 min each. A volume of 100 μl 5% BSA solution was added to each well and incubated at room temperature for 30 min. The primary antibody working solution was prepared with PBS to a concentration of 200 μg/ml, and 100 μl was added to each well and incubated overnight in a refrigerator. The 24-well cell culture dish was removed from the refrigerator and placed at room temperature to rewarm for 10 min, and PBST was added to the shaker and rinsed 3 times for 5 min. The CoraLite^®^488-conjugated 6*His His-Tag Mouse monoclonal antibody (Proteintech Group, Wuhan, China) was used as the secondary antibody at a concentration of 1:150, and 100 μl of the working solution of the secondary antibody was added and incubated at room temperature for 1 h. PBST was added to the shaker and rinsed 3 times for 5 min. A volume of 100 μl ready-to-use 4',6-diamidino-2-phenylindole (DAPI) working solution was added and incubated at room temperature for 4 min and rinsed with PBS 3 times for 5 min each time. The fluorescence signal was detected using a confocal microscope (ZEISS, LSM-800).

### Immunoaffinity purification of adenovirus from cell culture medium using immobilized sdAb

The conjugation of sdAb to the NHS-Activated Sefinose™ 4 Fast Flow (BBI, Beijing, China) was performed according to the manufacturer’s instructions. One microgram of purified sdAb was dissolved in 1 ml of coupling buffer (NaHCO_3_ and NaCl solution). One microliter of NHS-Sepharose FF was added to the gravity column, and 5 ml of 1 mM HCl was used to wash the column three times to remove the preservation solution. The antibody solution was added immediately after washing and shaken overnight at 4 °C for better coupling. The filtrate was collected the next day, the coupled Sepharose was rinsed with 5 ml of coupling buffer, and 1 ml of blocking buffer was used under shaking at room temperature for 4 h to block uncoupled agents. After removing the blocking buffer, the Sepharose was washed three times with 2 ml of coupling buffer. The recombinant human adenovirus HAd5 expressing enhanced green florescent protein (EGFP) (prepared in Department of Veterinary Public Health Faculty) was diluted in cell culture medium with a titer of 10^7^ vp/ml, added to the prepared immunoaffinity column, and combined for 30 min at room temperature. The bound virus was eluted with 2 M and 4 M NaCl solutions, and the eluent was added to the cultured HEK293A cells for reinfection. The presence of the recombinant virus in the eluent and the reinfected cells was visualized under a confocal microscope (ZEISS, LSM-800) by the expression of the EGFP.

## Results

### Bactrian camel immunization

**Fig. 2:**
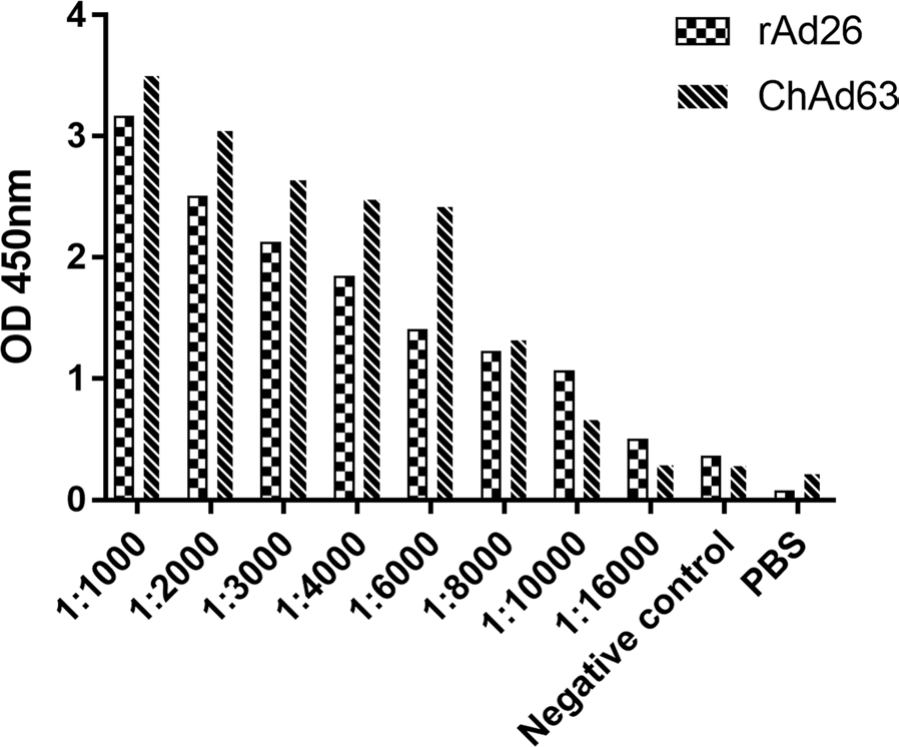
The serum antibody titer of immunized Bactrian camels was measured using ELISA. Polystyrene microplates were coated with rAd26 and ChAd63 viruses. The immune serum of the camel was diluted with PBS, basal serum was used as a negative control, and PBS was used as a blank control. The binding activity of each well was detected using an HRP-labeled goat anti-alpaca antibody at a 1:10,000 concentration and visualized with TMB solution. The plate was read at OD450 nm in a microplate reader

Basal serum collected before the immunization was used as a negative control, and anti-His-labeled goat anti-alpaca was used as a secondary antibody for detection in the ELISA. The OD450 nm value of basal serum was 0.375, and the OD450 nm value remained 2.1-fold higher than the negative control when the dilutions of immune sera specific for rAd26 and ChAd63 were 1:8000 and 1:10,000, respectively, as shown in Fig. 2. Therefore, we determined that the titers of immune serum were 1:8000 and 1:10,000.

### Construction and screening of Bactrian camel phage display antibody library

**Fig. 3:**
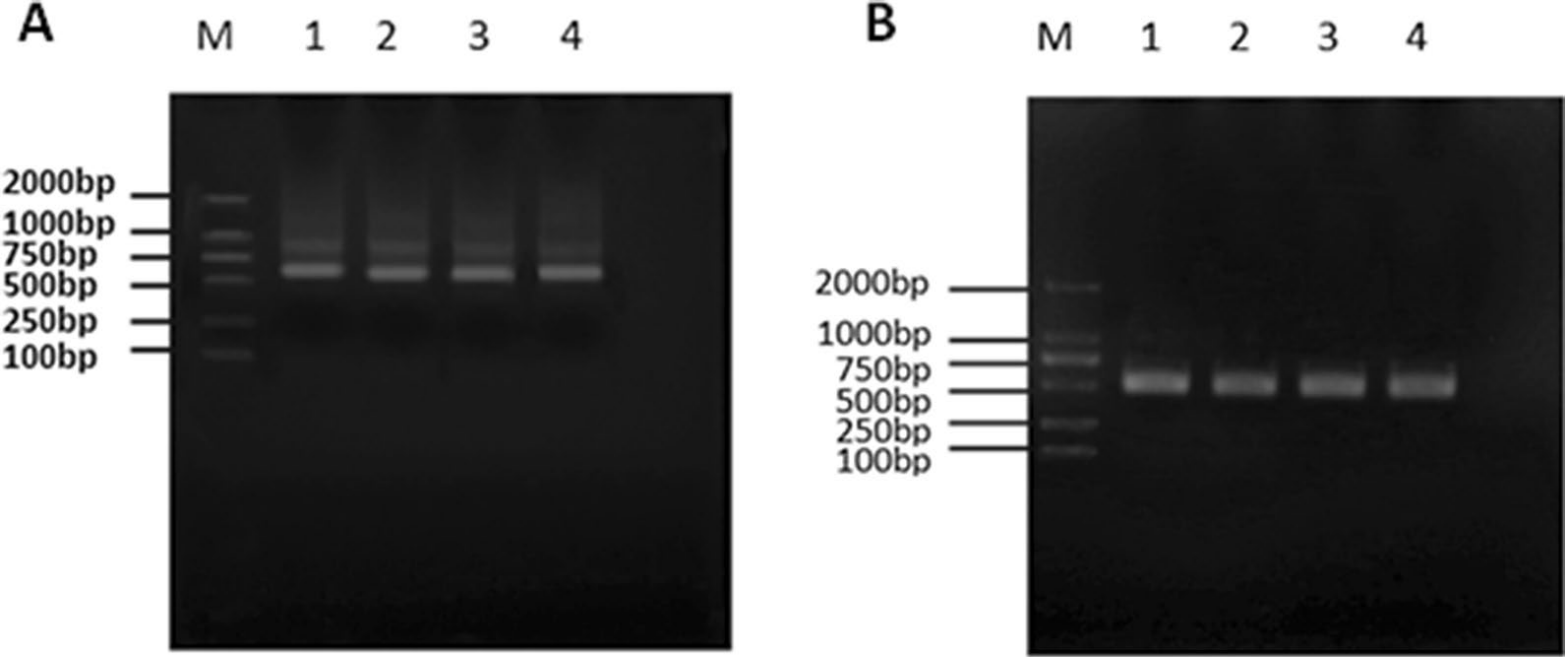
Amplification of VHH gene. cDNA was used as a template, and a fragment containing the leader sequence to the CH2 region of the IgG (900 bp for VH and 600 bp for VHH) was amplified with primers P_1_ and P_2_. The result is shown in Fig. 3(**a**). The 600 bp fragment was purified using 1% agarose gel electrophoresis and used as the template for the second round of PCR with primers P_3_ and P_4_, which anneal to the VHH FR1 and FR4 regions, respectively. The target bands of approximately 400 bp were successfully amplified, as shown in Fig. 3(**b**)

The VHH gene fragment was amplified from the cDNA obtained from the total RNA of camel PBMCs using nested PCR. The antibody leader peptide to the CH2 gene fragment was amplified from the cDNA in the first round of PCR using P_1_ and P_2_ as primers and detected by 1% agarose gel electrophoresis, as shown in Fig. 3a. A successfully amplified a gene fragment of approximately 600 bp was identified. The 600 bp product was recovered from the gel and used as the template for the second round of PCR. P_3_ and P_4_ primers were used to amplify the full-length VHH gene FR1 to FR4, and the fragment size was approximately 400 bp, as shown in Fig. 3b.

**Fig. 4:**
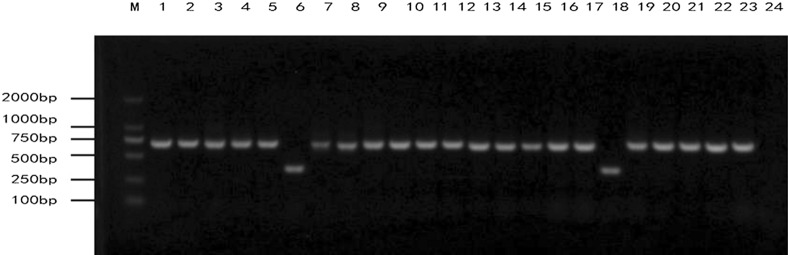
Identification of phage display antibody library. The electro-transformed TG1 bacterial solution from 10^1^ to 10^10^ with fresh 2 × YT/AG media was grown at 37 °C. The next day, the calculated volume of the phage antibody library was 1.8 × 10^9^. Twenty-four clones were randomly selected from the 2 × YT/AG plate for colony PCR identification. After 1% agarose gel electrophoresis, 22 clones with amplicons of 600 bp fragments were obtained, and we calculated that the recombination rate of the antibody library was 91.6%

The VHH gene fragment was digested and ligated into the pMECS vector and electro-transformed into competent *E. coli* TG1. The bacterial solution was diluted from 10^1^ to 10^10^, coated on 2 × YT-AG solid medium, and incubated overnight at 37 °C. The calculated phage repertoire with capacity was 1.8 × 10^9^. Twenty-four clones were randomly selected from the 2 × YT-AG solid medium for colony PCR identification. After 1% agarose gel electrophoresis, target bands of approximately 600 bp were obtained in 22 clones and are shown in Fig. 4. The correct insertion rate of the antibody fragment in the library was 91.6%.

**Fig. 5:**
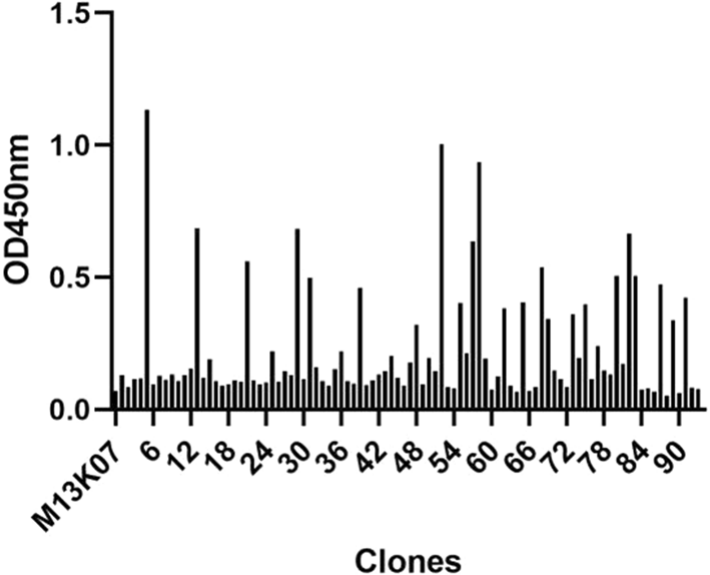
Detection of the immune activity of recombinant phages against adenovirus using phage ELISA. Recombinant phages prepared from 92 randomly selected clones were added to the microplates coated with adenovirus vector rAd26. The M13K07 helper phage was used as a negative control, and PBS was used as a blank control. The HRP/anti-M13 monoclonal antibody was used to detect the bound phage. The highest OD450 nm value of the recombinant phage was 1.133, and the lowest value was 0.052. The negative control was 0.07. Two positive clones with the highest OD450 nm value were selected and named sdAb 5 and sdAb 31

The adenovirus-specific recombinant phage was identified using phage ELISA. Ninety-two randomly selected recombinant phage clones were added to the microtiter plate coated with adenovirus vector rAd26, and HRP/anti-M13 monoclonal antibody was used to detect the bound phage. M13K07 helper phage was used as a negative control, and PBS was used as a blank control. The results are shown in Fig. 5. The measured OD405 nm value of the negative control was 0.07, and the highest value in the selected recombinant phage clone was 1.133. We identified 38 clones with OD450 nm values greater than 2.1-fold of the negative control. Two clones with the highest absorbance value were selected and named 5 and 31 for further identification.

### Expression and purification of sdAb

**Fig. 6:**
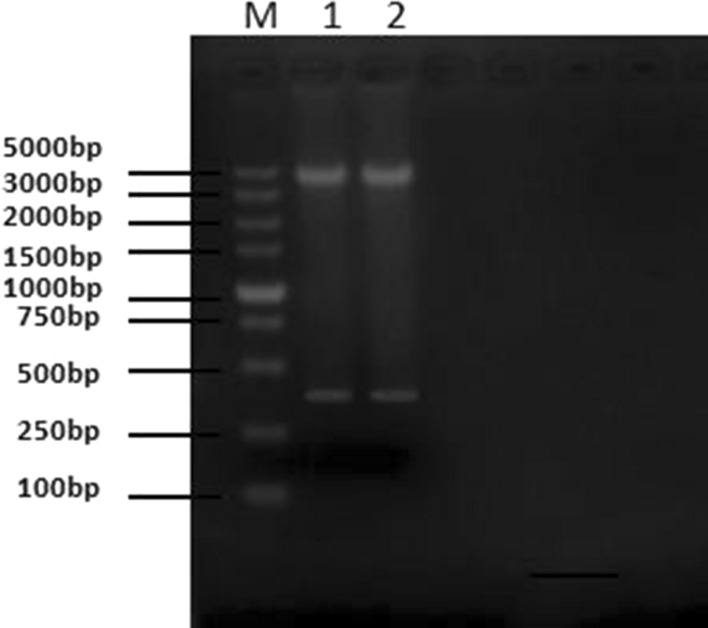
Recombinant plasmid digestion identification. The plasmids of the positive clones from phage ELISA were isolated, digested with *Nco*I and *Not*I, and detected using 1% agarose gel electrophoresis. A target band of approximately 400 bp was obtained

**Fig. 7:**
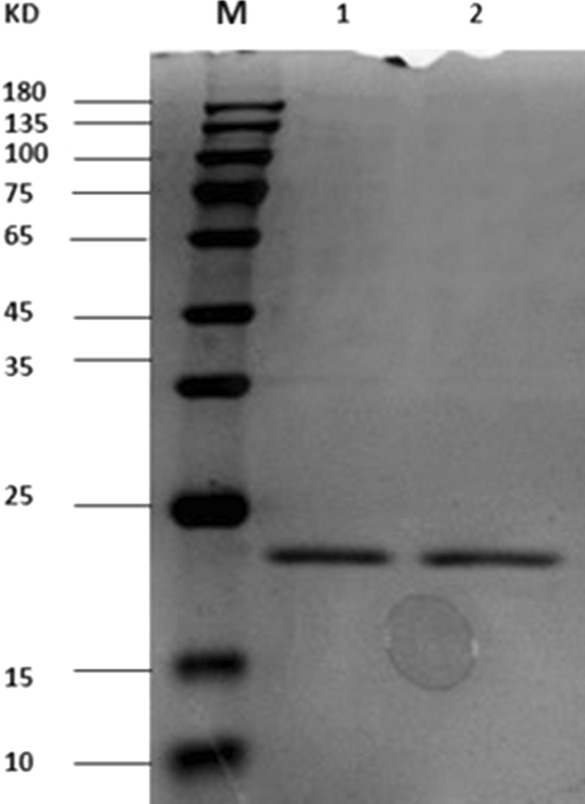
Expression and purification of the recombinant sdAbs. The plasmids of sdAb 5 and sdAb 31 from phage ELISA were isolated, and the VHH gene was digested with *Nco*I and *Not*I and ligated to pET-25b ( +) –SBP. The SDS-PAGE results showed that the recombinant sdAb 5 and sdAb 31 antibodies were expressed in a soluble form with an expected molecular weight of 20 kDa and could be successfully purified from Ni–NTA agarose

The plasmids of clones 5 and 31 were extracted, digested with the restriction enzymes *Nco*I and *Not*I, and detected using 1% agarose gel electrophoresis, as shown in Fig. 6. A target band of approximately 400 bp was obtained. The VHH gene fragment was ligated into pET-25b ( +) –SBP and transformed into *E. coli* BL21 (DE3). After IPTG induction, the recombinant sdAbs were expressed primarily in the supernatant of bacterial lysate by SDS-PAGE analysis (data not shown), and the sdAbs were applied to Ni–NTA Sefinose™ Resin for purification. The results of SDS-PAGE analysis on the purified sdAb showed a protein band at approximately 20 kDa, which was consistent with the expected result, as shown in Fig. 7.

### Binding activity and specificity of sdAb

**Fig. 8:**
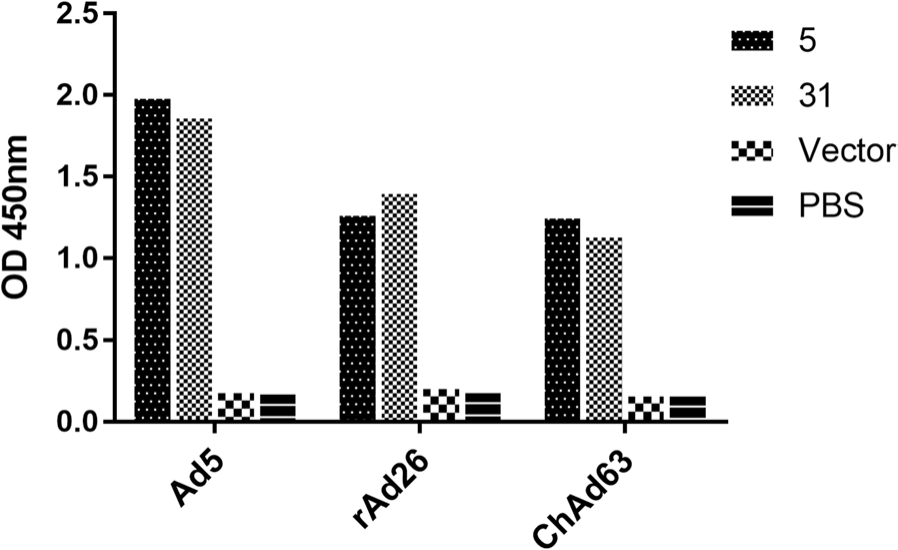
Binding activity and specificity were analyzed using indirect ELISA. The recombinant sdAbs 5 and 31 showed strong binding activity to human adenovirus Ad5, rAd26, and ChAd63 viruses in ELISAs at a concentration of 5 μg/ml. Protein extracts from *E. coli* transformed with the empty pET-25b ( +) vector were used as a negative control, and PBS solution was used as a blank control

**Fig. 9:**
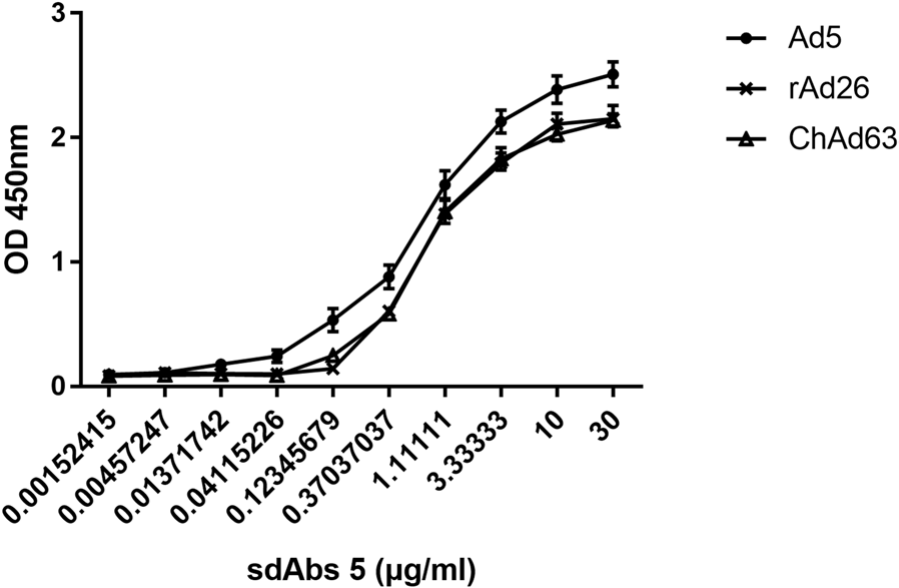
The binding activity of recombinant sdAbs 5 to three adenoviruses was analyzed using a typical ELISA. Recombinant sdAbs 5 showed binding activity to human adenovirus Ad5, rAd26 and ChAd63 viruses in ELISA at concentrations from 30 μg/ml to 0.00152415 μg/ml

The purified recombinant sdAb was used as the detection antibody to verify the binding activity to different adenoviruses in the ELISA. The recombinant sdAbs 5 and 31 showed strong binding activity to human adenovirus Ad5, rAd26, and ChAd63 viruses in ELISA s at a concentration of 5 μg/ml, as shown in Fig. 8, when PBS was used as a blank control, and *E. coli* BL21(DE3) cell lysate transformed with pET-25b ( +) empty vector was used as a negative control. The serial diluted recombinant clone 5 sdAb showed binding activity to human adenovirus Ad5, rAd26, and ChAd63 at a concentration of 30 μg/ml to 0.00152415 μg/ml, as shown in Fig. 9.

### Binding affinity of recombinant sdAb

**Fig. 10:**
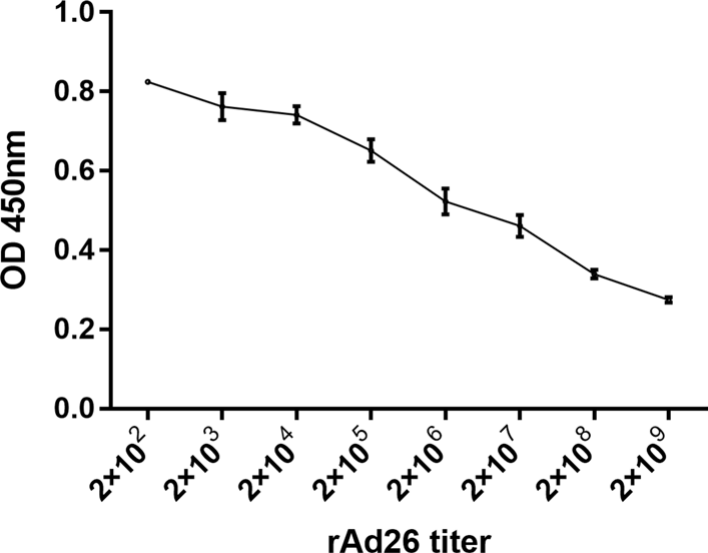
The affinity constants of recombinant sdAbs 5 to adenovirus rAd26 were determined by competitive ELISA. Affinity constants were expressed as the reciprocal of the adenovirus titer at 50% of the maximum OD450 nm value. The results showed that the 50% of the maximum OD450 nm value was 0.41225, and it can be found that the adenovirus titer at this point was about 5 × 10^8^ vp/ml. Therefore, the affinity constants of purified clone 5 sdAbs to adenovirus rAd26 were determined to be 2 × 10^–9^ by calculation

The affinity constants of purified clone 5 sdAbs to adenovirus rAd26 were determined by competitive ELISA. Affinity constants were expressed as the reciprocal of the adenovirus titer at 50% of the maximum OD450 nm value. The results showed that the maximum OD450 nm value was 0.8245, and 50% of the maximum OD450 nm value was 0.41225, and it can be found that the adenovirus titer at this point was about 5 × 10^8^ vp/ml, as shown in Fig. 10. Therefore, the affinity constants of purified clone 5 sdAbs to adenovirus rAd26 were determined to be 2 × 10^–9^ by calculation.

### Immunofluorescence assay

**Fig. 11:**
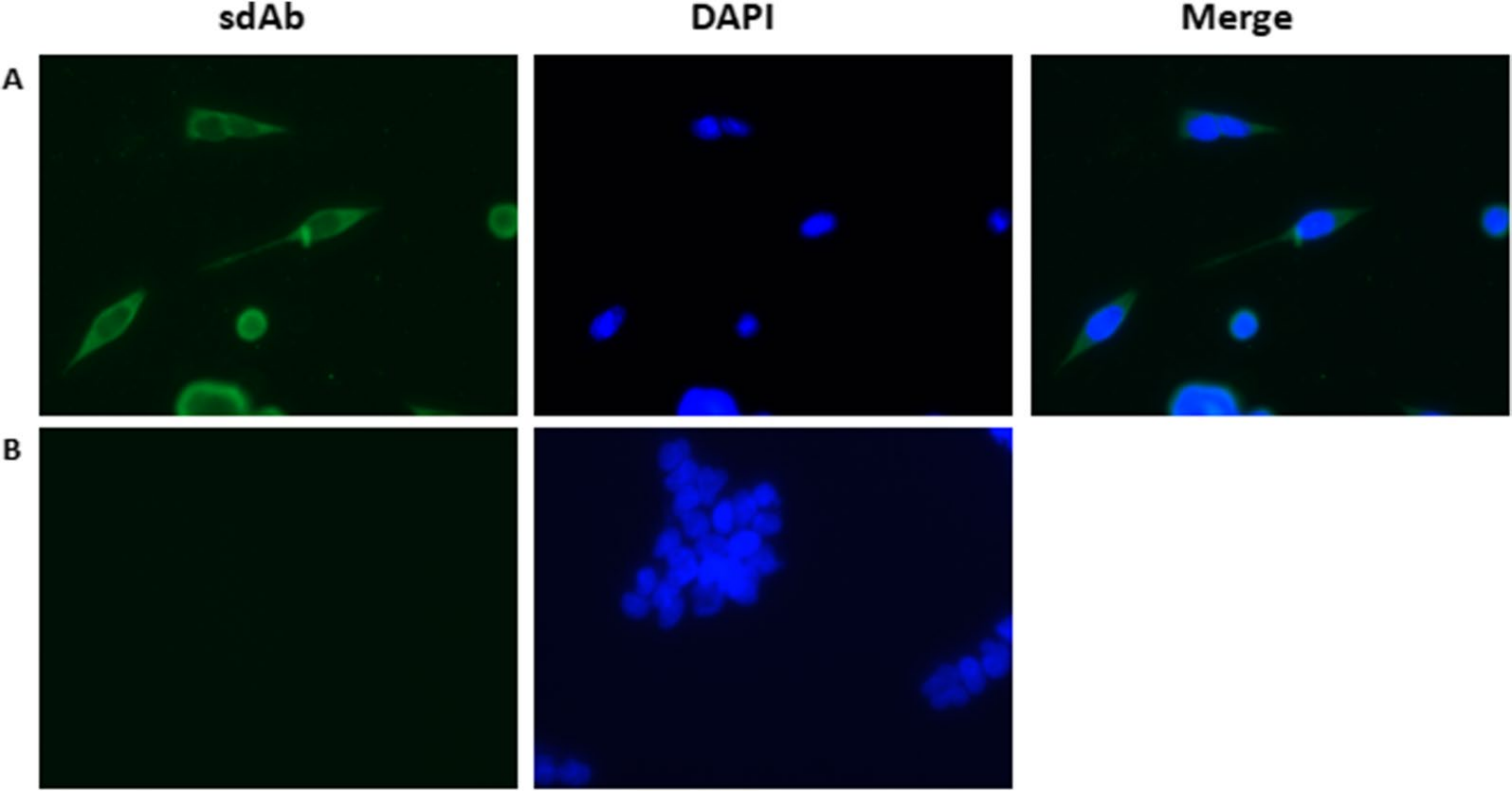
Immunofluorescence assay. Adenovirus-infected HEK293A cells were incubated with sdAb 5 antibody and visualized with CoraLite®488-conjugated 6*His His-Tag mouse monoclonal antibody. HEK293A cells uninfected with adenovirus rAd26 were used as a negative control. The cell nuclei were stained with DAPI. The fluorescent signal and images were obtained using confocal microscopy. The merged images showed the localization of adenovirus rAd26 in HEK293A cells (Fig. 11**A**), but there was no fluorescent signal in the uninfected HEK293A cells (Fig. 11**B**)

The results of the immunofluorescence assays are shown in Fig. 11. HEK293A cells were infected with rAd26 adenovirus, and recombinant sdAb 5 was used as the detection antibody then visualized with CoraLite®488-conjugated 6*His His-Tag mouse monoclonal antibody under an inverted fluorescence microscope. When using 488 nm as excitation light, we found a green fluorescence signal from the cells incubated with sdAb and primarily stained the cytoplasm of cells when merged with images of DAPI staining, as shown in Fig. 11A. However, we did not observe any fluorescence signal from the control group without incubation with sdAb, as shown in Fig. 11B. The results showed that sdAb specifically bound to adenoviruses-infected HEK293A cells and propagated.

### Immunoaffinity purification of adenovirus from cell culture media using immobilized sdAb

**Fig. 12:**
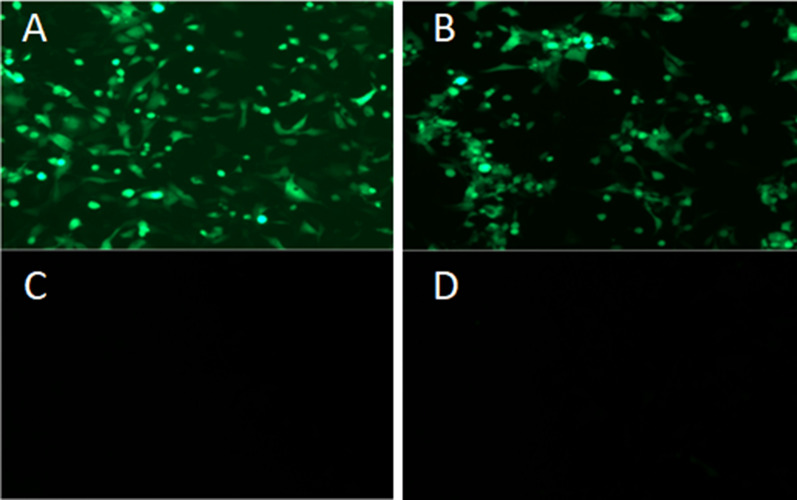
Immunoaffinity purification of adenovirus. The bound viruses were eluted with 2 M, 4 M NaCl solution and PBS, and the eluent was added to the cultured HEK293A cells for reinfection. A blank and negative control group were established. After inoculation, the cells were placed at 37 °C in a 5% CO_2_ incubator for 24 h to 48 h and observed and counted. **A** 2 M eluent group; **B** 4 M eluent group; **C** blank control group; **D** PBS buffer group. Green fluorescence was detected in the 2-M and 4-M NaCl solution eluent-infected cells, as shown in **A** and **B**, but we did not detect green fluorescence in PBS eluent-infected cells or non-infected cells, as shown in **C** and **D**. These results indicate that the immobilized sdAbs specifically bind to the adenoviruses and isolate them from the culture medium

The recombinant human adenovirus Ad5 expressing EGFP was diluted in cell culture medium at a titer of 10^7^ vp/ml, added to the prepared immunoaffinity column coupled with purified sdAb, and incubated for 30 min at room temperature. The bound virus was eluted with 2-M and 4-M NaCl solutions and PBS, and the eluent was added to the cultured HEK293A cells for reinfection. The presence of the recombinant virus in the eluent and reinfected cells was visualized under a confocal microscope by the expression of EGFP, as shown in Fig. 12. We observed green florescence in the 2-M and 4-M NaCl solution eluent-infected cells, as shown in Fig. 12A and B, but it was not detected in PBS eluent-infected cells or non-infected cells, as shown in Fig. 12C and D. These results indicated that the immobilized sdAb specifically bound to the adenoviruses and isolated the viruses from culture medium, and 2-M and 4-M NaCl solutions eluted the bound virus from the antibody.

## Discussion

Adenovirus is a linear double-stranded DNA that is enclosed in a capsid without an envelope structure. Human adenoviruses are one cause of acute respiratory infections in children. No safe and effective prevention methods have been developed, and there are more than 100 serotypes of human adenovirus. Because the diseases caused by various serotypes are different, and diverse disease severities are observed, research on related antibodies is of great significance for clinical diagnosis and therapy. Human adenoviruses have been studied in the basic virology field and are exploited as vectors for gene therapy, vaccination, and oncolytic therapy because Ads have three upstream regions that allow the insertion of foreign genes without affecting virus replication in the cell and because of the relatively thorough research on its structure and characteristics (Gao et al., 2019).

The outbreak of new coronavirus (2019-nCoV) infection poses a serious threat to global public health, and vaccination is an effective means to prevent viral infections. With the efforts of scientific researchers from different countries, adenovirus vector vaccines, such as Ad5, Ad26 and ChAdOx1, which encode the SARS-CoV-2 major antigen, have been developed and are already in clinical trials. The clinical trial showed that the adenovirus vector vaccine was a safe and effective strategy for the prevention and control of SARS-CoV-2 infection, and some of these vaccines received conditional marketing authorization for SARS-CoV-2 (Logunov et al., 2021; Madhi et al., 2021; Zhu et al., 2021).

Recombinant deficient adenoviruses have been widely used as a delivery system to express functional proteins for gene therapy. Two Ad5-based vectors expressing the tumor suppressor P53, named Advexin and Gendicine have generated a great deal of clinical data from their application. Advexin has been evaluated in different cancers, such as head and neck squamous cell carcinoma (HNSCC), colorectal cancer, and hepatocellular carcinoma (HCC), and Advexin demonstrated a consistent safety profile and clinical efficacy as a monotherapy and in combined modality regimens with chemotherapy and radiation (Senzer and Nemunaitis, 2009). Gendicine was approved in 2003 by the State Food and Drug Administration (SFDA) in China for intratumoral treatment of HNSCC in combination with chemotherapy (Tian et al., 2009).

Oncolytic adenoviruses have attracted much research interest due to their peculiar tumor selectivity, safety, and transgene-delivery capability. Adenoviruses armed with different immunostimulatory cytokines and chemokines have been developed. ONCOS-102 is an adenovirus armed with granulocyte macrophage colony stimulating factor (GM-CSF), and it showed a synergistic antitumor effect in humanized mice treated with the combination of ONCOS102 and pembrolizumab (Kuryk et al., 2019). Recent studies developed various adenoviruses armed with immune-activating ligands and bispecific T-cell engager (BiTE) molecules that were tested in clinical studies (Freedman et al., 2017; Malmstrom et al., 2010).

The recombinant adenovirus may be produced in cell culture and purified using ultracentrifugation in CsCl density gradients, followed by desalting using size exclusion chromatography (sepharose columns) or dialysis. However, the limitations of using CsCl density gradient ultracentrifugation are that it is time-consuming, the purification process is complex, and the virus activity is inconsistent between batches (Altaras et al., 2005). Immunoaffinity chromatography using a single domain antibody as the capturing ligand is a powerful tool for the single-step purification of recombinant virus and proteins from culture medium, with high purity and yields. Compared to conventional antibodies, single-domain antibodies lack the Fc domain of the antibody and avoid the existence of heavy and light contamination when used as immunoaffinity ligands and have been used widely in single-step processes (Ren et al., 2020; Verheesen et al., 2003).

The present study successfully constructed a single domain antibody phage display library with a capacity of 1.8 × 10^9^ by immunizing a Bactrian camel with adenoviruses. The phage antibody library was rescued, and the specific phages were enriched by adenoviruses. Twenty-two positive clones were screened, and two single domain antibodies with the highest binding activity and different sequences were selected for further expression and identification. Two recombinant sdAbs were solublely expressed in the bacteria and purified with Ni–NTA agarose. The two recombinant sdAbs specifically bound to adenoviruses in ELISA and immunofluorescence assays. We subsequently established a method for the immunoaffinity purification of adenovirus, which greatly saved purification time and cost. This method may be used to selectively capture Ads from vaccines or other virus culture mixtures, and it lays a foundation for the one-step immunoaffinity purification and identification of adenovirus.

## Data Availability

Please submit all requests to the authors.

## References

[CR1] Ahmadvand D, Rahbarizadeh F, Vishteh VK (2008). High-expression of monoclonal nanobodies used in the preparation of HRP-conjugated second antibody. Hybridoma.

[CR2] Altaras NE, Aunins JG, Evans RK, Kamen A, Konz JO, Wolf JJ (2005). Production and formulation of adenovirus vectors. Adv Biochem Eng Biotechnol.

[CR3] Antrobus RD, Coughlan L, Berthoud TK, Dicks MD, Hill AVS, Lambe T, Gilbert SC (2014). Clinical assessment of a novel recombinant Simian adenovirus ChAdOx1 as a vectored vaccine expressing conserved influenza A Antigens. Mol Ther.

[CR4] Barnes E, Folgori A, Capone S, Swadling L, Aston S, Kurioka A, Meyer J, Huddart R, Smith K, Townsend R, Brown A, Antrobus R, Ammendola V, Naddeo M, O'Hara G, Willberg C, Harrison A, Grazioli F, Esposito ML, Siani L, Traboni C, Oo Y, Adams D, Hill A, Colloca S, Nicosia A, Cortese R, Klenerman P (2012). Novel adenovirus-based vaccines induce broad and sustained T Cell responses to HCV in man. Sci Transl Med.

[CR5] Beghein E, Gettemans J (2017). Nanobody technology: a versatile toolkit for microscopic imaging, protein-protein interaction analysis, and protein function exploration. Front Immunol.

[CR6] Bett AJ, Haddara W, Prevec L, Graham FL (1994). An efficient and flexible system for construction of adenovirus vectors with insertions or deletions in early regions 1 and 3. Proc Natl Acad Sci USA.

[CR39] Burova E, Ioffe E (2005). Chromatographic purification of recombinant adenoviral and adeno-associated viral vectors: methods and implications. Gene Ther.

[CR7] Chang J (2021). Adenovirus vectors: excellent tools for vaccine development. Immune Netw.

[CR8] Coughlan L, Sridhar S, Payne R, Edmans M, Milicic A, Venkatraman N, Lugonja B, Clifton L, Qi C, Folegatti PM, Lawrie AM, Roberts R, de Graaf H, Sukhtankar P, Faust SN, Lewis DJM, Lambe T, Hill AVS, Gilbert SC (2018). Heterologous two-dose vaccination with Simian adenovirus and poxvirus vectors elicits long-lasting cellular immunity to Influenza Virus A in healthy adults (vol 29, pg 146, 2018). EBioMedicine.

[CR9] Farina SF, Gao GP, Xiang ZQ, Rux JJ, Burnett RM, Alvira MR, Marsh J, Ertl HC, Wilson JM (2001). Replication-defective vector based on a chimpanzee adenovirus. J Virol.

[CR10] Freedman JD, Hagel J, Scott EM, Psallidas I, Gupta A, Spiers L, Miller P, Kanellakis N, Ashfield R, Fisher KD, Duffy MR, Seymour LW (2017). Oncolytic adenovirus expressing bispecific antibody targets T-cell cytotoxicity in cancer biopsies. Embo Mol Med.

[CR11] Gao J, Mese K, Bunz O, Ehrhardt A (2019). State-of-the-art human adenovirus vectorology for therapeutic approaches. FEBS Lett.

[CR12] Gurwith M, Lock M, Taylor EM, Ishioka G, Alexander J, Mayall T, Ervin JE, Greenberg RN, Strout C, Treanor JJ, Webby R, Wright PF (2013). Safety and immunogenicity of an oral, replicating adenovirus serotype 4 vector vaccine for H5N1 influenza: a randomised, double-blind, placebo-controlled, phase 1 study. Lancet Infect Dis.

[CR13] Hu Y, Liu C, Muyldermans S (2017). Nanobody-based delivery systems for diagnosis and targeted tumor therapy. Front Immunol.

[CR14] Ison MG, Hayden RT (2016). Adenovirus. Microbiol Spectr.

[CR15] Kochanek S, Clemens PR, Mitani K, Chen HH, Chan S, Caskey CT (1996). A new adenoviral vector: replacement of all viral coding sequences with 28 kb of DNA independently expressing both full-length dystrophin and beta-galactosidase. Proc Natl Acad Sci USA.

[CR16] Kovesdi I, Hedley SJ (2010). Adenoviral producer cells. Viruses.

[CR17] Kuryk L, Moller AW, Jaderberg M (2019). Combination of immunogenic oncolytic adenovirus ONCOS-102 with anti-PD-1 pembrolizumab exhibits synergistic antitumor effect in humanized A2058 melanoma huNOG mouse model. Oncoimmunology.

[CR18] Lafaye P, Li TF (2018). Use of camel single-domain antibodies for the diagnosis and treatment of zoonotic diseases. Comp Immunol Microb.

[CR40] Lee D-S, Kim B-M, Seol D-W (2009). Improved purification of recombinant adenoviral vector by metal affinity membrane chromatography. Biochem Biophys Res Commun.

[CR19] Lewis AM, Rowe WP (1970). Isolation of two plaque variants from the adenovirus type 2-simian virus 40 hybrid population which differ in their efficiency in yielding simian virus 40. J Virol.

[CR20] Liebowitz D, Gottlieb K, Kolhatkar NS, Garg SJ, Asher JM, Nazareno J, Kim K, McIlwain DR, Tucker SN (2020). Efficacy, immunogenicity, and safety of an oral influenza vaccine: a placebo-controlled and active-controlled phase 2 human challenge study. Lancet Infect Dis.

[CR21] Logunov DY, Dolzhikova IV, Shcheblyakov DV, Tukhvatulin AI, Zubkova OV, Dzharullaeva AS, Kovyrshina AV, Lubenets NL, Grousova DM, Erokhova AS, Botikov AG, Izhaeva FM, Popova O, Ozharovskaya TA, Esmagambetov IB, Favorskaya IA, Zrelkin DI, Voronina DV, Shcherbinin DN, Semikhin AS, Simakova YV, Tokarskaya EA, Egorova DA, Shmarov MM, Nikitenko NA, Gushchin VA, Smolyarchuk EA, Zyryanov SK, Borisevich SV, Naroditsky BS, Gintsburg AL, Gam C-VVTG (2021). Safety and efficacy of an rAd26 and rAd5 vector-based heterologous prime-boost COVID-19 vaccine: an interim analysis of a randomised controlled phase 3 trial in Russia. Lancet.

[CR22] Madhi SA, Baillie VL, Cutland CL, Voysey M, Izu A (2021). Safety and efficacy of the ChAdOx1 nCoV-19 (AZD1222) Covid-19 vaccine against the B1351 variant in South Africa. Medrxiv.

[CR23] Malmstrom PU, Loskog AS, Lindqvist CA, Mangsbo SM, Fransson M, Wanders A, Gardmark T, Totterman TH (2010). AdCD40L immunogene therapy for bladder carcinoma–the first phase I/IIa trial. Clin Cancer Res.

[CR24] Molinier-Frenkel V, Lengagne R, Gaden F, Hong SS, Choppin J, Gahery-Segard H, Boulanger P, Guillet JG (2002). Adenovirus hexon protein is a potent adjuvant for activation of a cellular immune response. J Virol.

[CR25] Padlan EA (1996). X-ray crystallography of antibodies. Adv Protein Chem.

[CR26] Parks RJ, Chen L, Anton M, Sankar U, Rudnicki MA, Graham FL (1996). A helper-dependent adenovirus vector system: removal of helper virus by Cre-mediated excision of the viral packaging signal. Proc Natl Acad Sci USA.

[CR41] Peixoto C, Ferreira TB, Carrondo MJT, Cruz PE, Alves PM (2006). Purification of adenoviral vectors using expanded bed chromatography. J Virol Methods.

[CR27] Ren J, Zhang C, Ji FL, Jia LY (2020). Characterization and comparison of two peptide-tag specific nanobodies for immunoaffinity chromatography. J Chromatogr.

[CR28] Salvador JP, Vilaplana L, Marco MP (2019). Nanobody: outstanding features for diagnostic and therapeutic applications. Anal Bioanal Chem.

[CR29] Sebastian S, Lambe T (2018). Clinical advances in viral-vectored influenza vaccines. Vaccines-Basel.

[CR30] Senzer N, Nemunaitis J (2009). A review of contusugene ladenovec (Advexin) p53 therapy. Curr Opin Mol Ther.

[CR31] Shenk T (1996) Adenoviridae: the viruses and their replication. In: Fields BN, Knipe DM, Howley PM (eds) 3rd edn. vol 2, Lippincott-Raven, Philadelphia, pp 2111–2148 (Fields Virology)

[CR32] Tian G, Liu JL, Sui J, Zhou RM, Chen WH (2009). Multiple hepatic arterial injections of recombinant adenovirus p53 and 5-fluorouracil after transcatheter arterial chemoembolization for unresectable hepatocellular carcinoma: a pilot phase II trial. Anti-Cancer Drug.

[CR33] Trentin JJ, Yabe Y, Taylor GJS (1962). The quest for human cancer viruses: a new approach to an old problem reveals cancer induction in hamsters by human adenovirus. Science.

[CR34] Verheesen P, ten Haaft MR, Lindner N, Verrips CT, de Haard JJ (2003). Beneficial properties of single-domain antibody fragments for application in immunoaffinity purification and immuno-perfusion chromatography. Biochim Biophys Acta.

[CR35] Vincke C, Loris R, Saerens D, Martinez-Rodriguez S, Muyldermans S, Conrath K (2009). General strategy to humanize a Camelid single-domain antibody and identification of a universal humanized Nanobody Scaffold. J Biol Chem.

[CR36] Wang Q, Finer MH (1996). Second-generation adenovirus vectors. Nat Med.

[CR37] Wold WS, Toth K (2013). Adenovirus vectors for gene therapy, vaccination and cancer gene therapy. Curr Gene Ther.

[CR38] Zhu F, Jin P, Zhu T, Wang W, Ye H, Pan H, Hou L, Li J, Wang X, Wu S, Wang Y, Gou J, Huang H, Wu H, Wang X, Chen W (2021). Safety and immunogenicity of a recombinant adenovirus type-5-vectored COVID-19 vaccine with a homologous prime-boost regimen in healthy participants aged 6 years and above: a randomised, double-blind, placebo-controlled, phase 2b trial. Clin Infect Dis.

